# Prognostic significance of vascular endothelial cell growth factors -A, -C and -D in breast cancer and their relationship with angio- and lymphangiogenesis

**DOI:** 10.1038/sj.bjc.6603678

**Published:** 2007-03-13

**Authors:** R A A Mohammed, A Green, S El-Shikh, E C Paish, I O Ellis, S G Martin

**Affiliations:** 1 Department of Clinical Oncology, University Hospitals, City Hospital Campus, University of Nottingham, Hucknall Road, NG5 1PB, Nottingham, UK; 2 Histopathology Departments, University Hospitals, City Hospital Campus, University of Nottingham, Hucknall Road, NG5 1PB, Nottingham, UK

**Keywords:** breast cancer, vascular endothelial cell growth factors, VEGF-A, VEGF-C, VEGF-D, lymphangiogenesis, angiogenesis

## Abstract

Vascular endothelial cell growth factors (VEGF)-A, -C and -D have potent angio and lymphangiogenic functions in experimental models, although their role in the progression of human breast cancer is unclear. The aims of the current study were to examine the relationship between the expression of the aforementioned growth factors with the angio and lymphangiogenic characteristics of breast cancer, and to assess their suitability as potential prognostic factors. Paraffin-embedded sections of 177 primary invasive breast cancer, with complete clinical follow-up information for 10 years, were stained for VEGF-A, -C, -D, podoplanin and CD34 using standard immunohistochemical approaches. The expression of the growth factors was correlated with clinicopathological criteria and patients’ survival. Lymph vessel density (LVD) and microvessel density (MVD) were assessed and correlated with expression of the growth factors. Vascular endothelial cell growth factor-A, -C and -D were highly expressed in 40, 37 and 42% of specimens, respectively. High expression of VEGF-A and - C, but not of -D, was associated with a higher LVD (*P*=0.013 and *P*=0.014, respectively), a higher MVD (*P*<0.001 and *P*=0.002, respectively), the presence of lymph node metastasis (*P*<0.001 and *P*<0.001, respectively), distant metastasis (*P*=0.010 and *P*=0.008, respectively) and a shorter Overall Survival (*P*=0.029 and 0.028, respectively). In conclusion, breast cancers that express high levels of VEGF-A and -C are characterised by a poor prognosis, likely through the induction of angio and lymphangiogenesis. Examination of expression of VEGF-A and -C in breast cancer may be beneficial in the identification of a subset of tumours that have a higher probability of recurrence and metastatic spread.

Angiogenesis, the formation of new blood vessels, and lymphangiogenesis, the formation of new lymphatics, are complex processes in which different signalling systems work together; the main system being the vascular endothelial cell growth factor (VEGF) family of proteins and the associated receptors. This system is composed of a family of glycoprotein growth factors; VEGF-A, -B, -C, -D, -E and the placenta growth factor (PLGF) (Ferrara and Davis-Smyth, 1997). They bind selectively, with different affinities to three receptors that belong to the superfamily of receptor tyrosine kinases; VEGFR-1, -2 and -3. Both VEGFR-1 and VEGFR-2 are expressed on the surface of blood endothelial cells (BECs). Vascular endothelial cell growth factor receptor-3 is expressed throughout both blood and lymphatic vasculature during embryological development and in tumour tissues, however in normal adult tissues it is restricted to lymphatic endothelial cells (LECs) (Partanen and Paavonen, 2001).

Vascular endothelial cell growth factor-A, a multifunctional cytokine secreted by a large variety of cells, is known to play an essential role in vasculogenesis and angiogenesis (Fox *et al*, 2001). During transcription, it undergoes alternative splicing, yielding multiple mature glycoprotein isoforms with different amino-acid lengths (VEGF-A165, -A121, -A189 and -A206) that are capable of inducing proliferation and migration of endothelial cells (ECs) (Ferrara and Bunting, 1996). Expression of VEGF-A has been found to be upregulated and suggested to be associated with progression of certain types of human tumours such as lung (Han *et al*, 2001), eosophageal (Kleespies *et al*, 2005) and colorectal cancers (Des Guetz *et al*, 2006). Studies on human breast cancer reported a significant relationship between high expression of VEGF-A and tumours with higher proliferation rate, negative oestrogen receptor status (Fuckar *et al*, 2006) and poor prognosis (Linderholm *et al*, 1999).

The role of VEGF-A as a potent angiogenic factor in malignant tumours is well established, but it has long been thought that it had no influence upon lymphangiogenesis. It has recently been reported, however, that VEGF-A can induce lymphangiogenesis as well as angiogenesis (Nagy *et al*, 2002). The relationship between the expression of VEGF-A and lymphangiogenesis in breast cancer has not been studied and is a component of the current study.

VEGF-C and -D have potent lymphangiogenic functions through stimulation of VEGFR-3 on the surface of LECs. They are produced in pro–prepeptide dimers that undergo proteolysis in the extracellular matrix (ECM) to a mature form that has much higher affinity to VEGFR-3, inducing lymphangiogenesis, but can also bind VEGFR-2, inducing angiogenesis (Joukov *et al*, 1996). Overexpression of VEGF-C and -D in experimental tumour models has been found to be significantly associated with the formation of new lymphatics (Skobe *et al*, 2001; Stacker *et al*, 2001; Von Marschall *et al*, 2005), however, their effect in human cancers is a matter of controversy with some studies finding a significant association with tumour angiogenesis, lymphangiogenesis, lymph node (LN) status and prognosis (Nakamura *et al*, 2003a, 2005) and others no relationships (Currie *et al*, 2004).

The mainstay of assessment of tumour vascularity has been counting the number of immunohistochemically identified microvessels in vascular hot spots, known as the microvessel density (MVD) (Weidner *et al*, 1992), using vascular markers such as factor VIII-related antigen, CD34 and CD31, with a recent review recommending procedures that should be followed for the assessment of MVD in breast cancer (Fox and Harris, 2004). The assessment of lymphatic characteristics in malignant tumours has historically been difficult owing to the lack of availability of lymphatic-specific markers. Such markers have recently been characterised and become commercially available. Markers include podoplanin, D2-40, LYVE-1 and Prox-1. The count of positively stained vessels per tumour area, lymph vessel density (LVD), has been used to assess lymphangiogenic characteristics in tumour specimens (Rodriguez-Niedenfuhr *et al*, 2001; Sahni *et al*, 2005). High MVD (Weidner *et al*, 1991; Bevilacqua *et al*, 1995; Toi *et al*, 1995b; Choi *et al*, 2005) and high LVD (Choi *et al*, 2005) in breast cancer have been reported to be associated with more aggressive tumour behaviour and poorer survival.

Although a number of studies have examined the relationship between the expression of each of the VEGFs with patient prognosis, angiogenic and lymphangiogenic characteristics in breast cancer, examination of the expression of all three, together, in a well-characterised series of breast cancers with long-term follow-up has not been conducted. The aims of the current study were to: (a) investigate the expression of VEGF-A, -C and -D in human breast cancer via immunohistochemistry, (b) examine the tumour lymphatic and vascular characteristics by counting lymph vessels/area to determine LVD and counting blood vessels via the Chalkley method to assess MVD and (c) conduct correlations between expression of the growth factors in relation to patient clinicopathological data, survival, lymphangiogenesis and angiogenesis to examine the role that they play in the regulation of such processes and in the progression of breast cancer.

## MATERIALS AND METHODS

### Patients and tissue samples

One hundred and seventy-seven paraffin-embedded archival specimens of primary invasive breast cancer were retrieved from the Department of Histopathology, Nottingham University Hospitals, City Hospital Campus. The median age of patients at time of diagnosis was 57 years (range 32–70 years). Fifty-one patients (28.2%) were younger than 50 years. Sixty-five (36.7%) of the specimens were <1.5 cm. At the time of primary diagnosis, 52 (29.4%) patients had positive LNs. Most of the tumours were stage I (*n*=121, 68.4%) and stage II (*n*=43, 24.3%) disease. Complete clinical follow-up information was available for all 177 patients, with ethical approval obtained for analysis from Nottingham Local Research Ethics Committee (REC C2020313). The median follow-up period was 96 months. Forty-one patients developed regional recurrence by the time of the last follow-up and 16 patients died from breast cancer.

### Immunohistochemistry

Staining with two lymphatic markers, D2-40 (SIGNET, 730-16) and podoplanin was carried out on paraffin-embedded blocks of tonsil, LN and breast cancer tissues. Recently, however, Schacht *et al* (2005) found that D2-40 and podoplanin detect the same protein on LECs. As both markers showed the same intensity of staining, and the same pattern, either can be used to generate the same results.

A representative, paraffin-embedded section from each specimen was stained with Podoplanin (polyclonal Ab, AngioBio, CA, USA, 11-003, 1 : 100 dilution), CD34 (polyclonal Ab, SEROTEC, Oxford, UK, MCAP547, 1 : 500 dilution), VEGF-A (monoclonal Ab, LAB VISION, CA, USA, RM-9128-S, 1 : 100 dilution), VEGF-C (polyclonal Ab, ZYMED, CA, USA, 18-2255, 1 : 75 dilution) and VEGF-D (monoclonal Ab, R&D, Abingdon, UK, MAB286 in 1 : 400 dilution). Briefly, 4-*μ*m-thick sections were deparaffinised with xylene and rehydrated. Antigen retrieval for CD34, VEGF-A, -C and -D was achieved by incubating sections in 0.01 mol l^−1^ sodium citrate buffer (pH 6.0) in a 800 W microwave for 20 min; 10 min at 800 W then 10 min at 100 W. Podoplanin did not require antigen retrieval. Endogenous hydrogen peroxidase (H_2_O_2_) reactivity was blocked with 3% H_2_O_2_. Nonspecific reactions were blocked by use of normal swine serum for 20 min. Sections were incubated for 1 h at room temperature for all primary antibodies except for VEGF-D that was incubated overnight at 4°C. After washing, sections were treated with commercial biotinylated secondary anti-immunoglobulin, followed by avidin coupled to biotinylated horseradish peroxidase, at room temperature, according to the manufacturer's instructions using streptABC kit (StreptABComplex/HRP Duet, Mouse/Rabbit kit, DAKO Corporation, Denmark, K0492). Immunohistochemical reactions were developed with 3,3′ diaminobenzidine as the chromogenic peroxidase substrate (DAKO, K3468). Sections were then counterstained with Myer's haematoxylein, dehydrated, fixed in xylene and mounted with DPX. Sections from placenta were used as a positive control, for VEGFs and CD34, and sections from LN were used for podoplanin. For negative controls, sections were stained using the same procedure after omitting primary antibody.

### Evaluation of growth factor expression

Expression of VEGF-A, -C and -D was assessed semiquantitatively using an immunohistochemical score (*H* score). Staining intensity was given four grades: none (0), weak (1), moderate (2) and strong (3). *H* score was calculated by multiplying the percentage of positive carcinoma cells by the staining intensity, this gave an *H* score ranging from 0 to 300. The median of the score was selected as the cutoff level according to which tumours were categorised into low- and high-expressing tumours. Median values were 160 for VEGF-A, 140 for VEGF-C and 130 for VEGF-D. This method, in selecting an appropriate cutoff level, has been used in previous studies (Yang *et al*, 2002).

### Assessment of LVD

The commonly used method for assessment of density of lymph vessels in tumour has been counting the number of immunohistochemically identified lymphatics in the most vascularised tumour areas; hot spots (Giorgadze *et al*, 2004; Koskinen *et al*, 2005; Nakamura *et al*, 2005). In the present study, a modified procedure was used, where LVD was assessed by counting all lymph vessels in the whole tumour section, using × 100 magnification with a surface area of 3.46 mm^2^. Although easier to use the × 200 magnification to assess LVD, the strong staining of lymphatics allowed easy recognition of vessels at × 100, especially when examining the whole tumour section. The sum of lymph vessels was divided by the sum of the surface area of all counted fields to adjust LVD per mm^2^. Lymph vessel density was presented as number of lymph vessels per mm^2^. Although more labour intensive, such methodology allows a more accurate assessment of lymphatic density. The distribution of lymphatics and prevalence of lymphovascular invasion will be the focus of a separate report.

### Assessment of MVD

Sections stained with CD34 were used for the evaluation of MVD using the Chalkley counting method. Each section was first scanned at low-power magnification (× 40) to select the most vascularised areas; three hot spots were selected. Two authors first examined 10% of specimens to agree on which fields to be used as hot spots. A 25-point Chalkley eyepiece graticule was applied to each hot spot and oriented to permit the maximum number of points to hit on, or within the areas of immunohistochemically highlighted microvessel using × 200 magnification. A Chalkley count for an individual tumour was taken as the mean value of the three graticule counts (Fox and Harris, 2004).

For assessment of LVD, MVD and expression of VEGFs, a second investigator also blinded to the patients’ clinical characteristics and survival data, independently assessed 20% of sections. A *κ*>0.90 was obtained, indicating a very good correlation between observers.

### Statistical analysis

Four levels of statistics were performed using SPSS for windows, version 13: (1) Mann–Whitney tests were conducted to compare means of LVD and MVD (as a continuous data) between different clinicopathological groups and (2) specimens were divided into two categories according to the median values of LVD and MVD. The association between expression of VEGFs and LVD, MVD and clinicopathological criteria was evaluated in univariate analysis using a 2 × 2 table and *χ*^2^ test, (3) survival analysis of disease-free interval (DFI) and overall survival (OS) was accomplished using the Kaplan–Meier method and the statistical significance of differences in the cumulative survival curves between groups was evaluated by the long-rank test. Multivariate survival analysis was performed using the Cox's proportional hazard method and (4) the Kappa (*κ*) agreement test was used to assess agreement between observers. All statistical analyses were two sided with significance defined as *P*<0.05.

## RESULTS

### Expression of VEGFs

**Figure 1:**
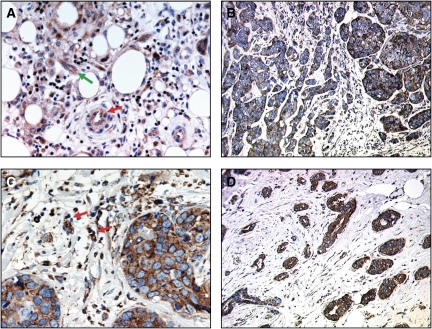
(**A**) Peritumoural area of a breast carcinoma section stained with VEGF-A showing positive staining in the endothelial cells (red arrow) and in the macrophages (green arrow), × 400. (**B**) Grade-III infiltrating breast carcinoma showing heterogeneous staining intensity for VEGF-A in the same specimen; tumour sheets with weak staining intensity on the left and others with strong staining intensity on the right, × 200. (**C**) VEGF-C-stained tumour section showing strong positive staining in the tumour-associated macrophages (red arrow) and in the tumour cells, × 400. (**D**) Grade-II infiltrating breast carcinoma showing strong positive staining with VEGF-D, × 100.

Expression of all three VEGFs showed a positive cytoplasmic staining in the breast cancer cells, with granular and heterogeneous staining in some specimens. Positive staining for VEGF-A was detected in the normal mammary epithelial cells adjacent to tumour, in the ECs and in the tumour-associated macrophages (Figure 1A). Seventy-seven (40%) specimens of breast carcinomas showed high expression of VEGF-A, with stronger staining intensity found in the invasive component of the tumour and vascular emboli. In many specimens, the staining intensity was heterogeneous among tumour areas (Figure 1B). Consistent with the findings of others (Nakamura *et al*, 2003a; Jennbacken *et al*, 2005), positive staining with VEGF-C and -D was observed in the normal mammary epithelial cells, tumour-associated macrophages, stromal cells and ECs (Figure 1C)Vascular endothelial cell growth factor-D showed positive staining in the vascular smooth muscle fibres. Sixty-seven (37%) and 91 (44%) specimens showed strong staining with VEGF-C and -D, respectively (Figures 1C and D).

The relationship between expressions of the three growth factors was analyzed. Specimens with high expression of VEGF-A found to be significantly associated with high expression of VEGF-C (*P*=0.046) but not with expression of VEGF-D (*P*=0.354). There was, however, a significant association between expression of VEGF-C and -D where 64% of cases with high expression of VEGF-C had high expression of VEGF-D (*P*=0.001), Table 1.

### Relationships amongst expression of VEGFs, angiogenesis and lymphangiogenesis

**Figure 2:**
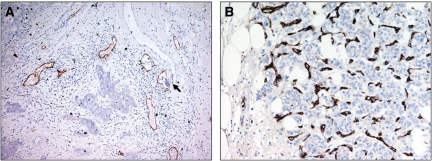
(**A**) Infiltrating duct carcinoma of the breast stained with podoplanin showing multiple lymphatics at the peritumoural area with a lymphatic invasion in one of the vessels (arrow), × 200. (**B**) Infiltrating duct carcinoma of the breast stained with CD34 showing high MVD, × 200.

**Figure 3:**
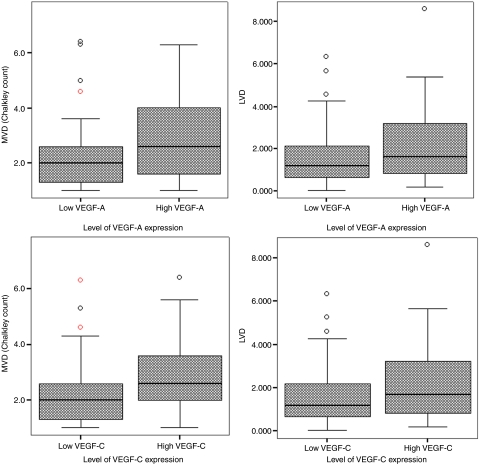
Box plots showing relationships between expression of VEGF-A and -C with MVD and LVD, with the middle line in each box representing the median value.

Lymph vessel density ranged from 0.015 mm^−2^ to 8.59 mm^−2^ with a mean of 1.7 mm^−2^±0.1 and a median of 1.37. Fifty-five (31.1%) specimens were characterised with a high LVD, Figure 2A. MVD ranged from 1.0 to 7.6 with a mean of 2.5±0.09 and a median of 2.3. Seventy-six (42.9%) specimens were characterised with high MVD, Figure 2B. The relationship amongst VEGF-A, -C and -D expression, MVD and LVD are summarised in Table 2. Tumours with high expression of VEGF-A were significantly associated with both a higher MVD (*P*<0.001) and higher LVD (*P*=0.013). Such tumours had a mean MVD of 2.9±0.15 and a mean LVD of 1.5±0.11 compared to 2.2±0.11 and 2.0±0.19 for specimens with low VEGF-A expression respectively. Similar findings were found with tumours with high expression, of VEGF-C. Such tumours had a mean MVD of 2.8±0.17 and a mean LVD of 2.2±0.22 compared with 2.3±0.11 and 1.5±0.11 for patients with low VEGF-C expression respectively (*P*=0.002 for MVD and *P*=0.014 for LVD), such relationships are illustrated using box plots in Figure 3. No statistical significant association was found between expression of VEGF-D and LVD or with MVD.

### Relationships between expression of VEGFs and clinicopathological criteria

A comparison between low and high growth factor expressing tumours was conducted to examine for potential associations with clinicopathological characteristics (Table 2). High expression of VEGF-A was significantly associated with tumours larger than 1.5 cm (*P*=0.038) and also with grade III tumours (*P*<0.001). Sixty-four percent of breast carcinomas with negative oestrogen receptors (ER) and 55% of those with negative progesterone receptor (PR) status express significantly higher levels of VEGF-A than hormonal receptor positive carcinomas (*P*<0.001 and *P*=0.004, respectively). A significant association was also found with the presence of axillary LN (*P*<0.001) and presence of distant metastasises (*P*=0.010) but not with patients’ age.

A significant positive relationship between VEGF-C expression and presence of LN metastasis (*P*<0.001) was detected. Tumours larger than 1.5 cm had a significantly higher level of VEGF-C expression (*P*=0.023) but unlike VEGF-A, no association was found in relation to the tumour grade (*P*=0.214) or with the hormonal receptor status (*P*=0.865 for ER and *P*=0.624 for PR).

No significant association was found between expression of VEGF-D and any of the clinicopathological criteria. However, a larger proportion of tumours with high expression with VEGF-D (54%) had LN metastasis compared to low expressing tumours (42%) but was not statistically significant (*P*=0.187).

### Prognostic significance of growth factor expression

**Figure 4:**
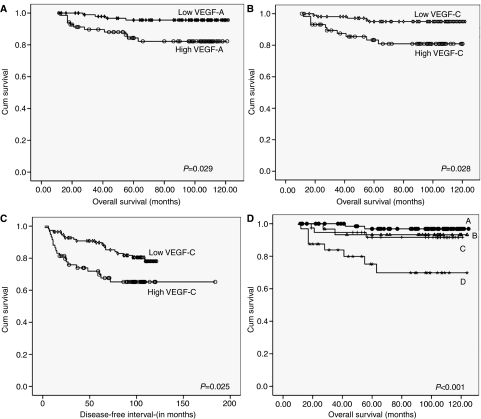
Associations between expression of VEGF-A and -C with patient prognosis using Kaplan–Meier method. High expression of VEGF-A is significantly associated with decreased OS (**A**). High expression of VEGF-C is significantly associated with decreased OS (**B**) and DFI (**C**). Comparison between survival in four groups: group A (low expression of both VEGF-A and VEGF-C, group B; (high expression VEGF-A), group C; (high expression of VEGF-C) and group D; (high expression of both VEGF-A and VEGF-C) showing that group D has the worst prognosis.

Kaplan–Meier analysis for OS and DFI were conducted to investigate whether expression of VEGF-A, -C and -D had any prognostic significance. From univariate analysis, high expression of VEGF-A was significantly associated with shorter OS (*P*=0.029), Figure 4A. High expression of VEGF-C was significantly associated with both shorter OS (*P*=0.025 and DFI (*P*=0.028) (Figure 4B and C), however VEGF-D was neither associated with DFI nor OS. Only VEGF-C retained significance upon multivariate analysis, when adjusted for tumour size, tumour grade and patient age (*P*=0.047, hazard ratio 2.854; 95% confidence interval 1.016–8.015).

Tumours were subsequently recategorised according to expression of VEGF-A and -C into four groups; group (A); tumours with low expression of both VEGF-A and -C, group (B); tumours with high expression of VEGF-A, group (C); tumours with high expression of VEGF-C and group (D); tumours with high expression of both VEGF-A and -C. On survival analysis, tumours with high expression of both VEGF-A and -C had the shortest OS when compared with the other three groups (*P*<0.001) (Figure 4D). The prognostic significance of LVD and MVD will be a focus of a separate report examining the role of lymphatic distribution, density and lymphovascular invasion in relationship to clinicopathological criteria of tumours and patients’ survival.

## DISCUSSION

The present report studied the expression of VEGF-A, -C and -D in breast cancer and correlated results with the lymphangiogenic and angiogenic characteristics of the tumours.

Vascular endothelial growth factor-A was originally identified as an EC-specific growth factor that induces angiogenesis and increases vascular permeability. It was found to be expressed in normal human tissues including liver, kidney, adrenal glands, lung and stomach (Ferrara and Davis-Smyth, 1997). In the study of Brown *et al*, (1993), VEGF-A mRNA was detected in the breast cancer cells whereas the corresponding proteins were found in both tumour cells and the ECs, indicating that VEGF-A is secreted by tumour cells then undergoes processing in the ECM to be entrapped by the receptors on the surface of the ECs. In the current report, and in agreement with such previous findings, positive staining for VEGF-A was detected in the ECs, in the normal epithelial mammary duct cells as well as in the tumour cells.

Similar to findings reported by other studies on breast cancer (Toi *et al*, 1995a; Hicklin and Ellis, 2005) and on other tumour types (Han *et al*, 2001), tumours with high expression of VEGF-A were characterised by a significantly higher MVD. This may be due to the multiple effects of VEGF-A on ECs. Not only is it a potent mitogenic factor but it also stimulates ECs to secrete cytokines essential for cell migration and sprouting of new vessels. It has long been supposed that VEGF-A had no association with lymphangiogenesis, however, in two recent experimental studies, VEGF-A overexpression was found to induce formation of new lymph vessels and dilatation of pre-existing ones (Nagy *et al*, 2002; Kunstfeld *et al*, 2004). This action was found to be mediated through stimulation of VEGFR-2, *α*1*β*1 and *α*2*β*1 integrins on the surface of LECs (Hong *et al*, 2004). Certain studies have reported a significant relationship between VEGF-A expression and lymphangiogenesis in malignant lymphoma (Kadowaki *et al*, 2005) and in lung carcinoma (Niki *et al*, 2000). The current study is the first, to our knowledge, to report such a relationship in human breast cancer, in that a significant association between VEGF-A expression and high LVD was observed. Such results suggest an important role for VEGF-A in the induction of both angiogenesis and lymphangiogenesis in breast cancer.

High expression of VEGF-A was found to be associated with tumours larger than 1.5 cm in size. This is in agreement with a previous study on breast cancer (Toi *et al*, 1995a) and one on lung cancer (Niki *et al*, 2000), where they found that high expression of VEGF-A was not only associated with larger tumours but also with larger metastatic deposits, likely through the growth factor inducing a rich vascular network, and a correspondingly more nutritious environment for tumour growth. The current study also found that such tumours behaved more aggressively, as they were significantly associated with the presence of LN metastasis (*P*<0.001), distant metastasis (*P*=0.01) and a poorer survival (*P*=0.029). These findings are similar to others, both in breast cancer and other tumour types (Toi *et al*, 1995b; Linderholm *et al*, 1999; O-Charoenrat *et al*, 2001). Although the effect of VEGF-A expression can be mediated by an increased vascular network, it should be noted that the growth factor has also been shown to increase breast cancer cell survival through direct action on VEGFR-2 that has been found to be expressed on the surface of the breast cancer cells. Vascular endothelial cell growth factor-A production by, and VEGFR-2 activation on, the surface of tumour cells indicates the presence of a distinct autocrine signalling loop that enables breast cancer cells to promote their own growth and survival by activation of VEGFR-2 (Weigand *et al*, 2005). The findings in this study support others that have reported the important role of VEGF-A in the progression of breast cancer; a role mediated through angiogenesis and promotion of tumour cell survival, and add the induction of lymphangiogenesis as another possible mechanism.

Similar to previous reports (Gunningham *et al*, 2000), the present study detected positive staining for VEGF-C and -D in normal mammary epithelial cells at a weaker intensity than in the surrounding invasive component suggesting a role in remodelling lymphatic and blood vasculature in mammary stroma during the menstrual cycle. It is known that the normal human mammary gland undergoes a well-defined sequence of changes in both epithelial and stromal compartments during the menstrual cycle, with increased angiogenesis during the ovulatory phase under effect of hormonal changes (Ferguson *et al*, 1992; Weinstein *et al*, 2005).

Using animal models, VEGF-C induced formation of new lymph vessels in the chick chorioallantoic membrane (Oh *et al*, 1997), induced lymphangiogenesis around islet of Langerhans in the pancreas which, normally, are not surrounded with lymphatics (Mandriota *et al*, 2001), induced hyperplasia and dilatation of the dermal lymphatics when overexpressed in transgenic mice (Jeltsch *et al*, 1997) and increased LVD and LN metastasis in xenografted breast cancer (Skobe *et al*, 2001). The present study reports a significant association between expression of VEGF-C and LVD similar to other findings reported in breast cancer (Nakamura *et al*, 2005) and other tumour types (Yonemura *et al*, 1999; Onogawa *et al*, 2004). This is mostly likely due to the stimulatory effect of VEGF-C on VEGFR-3 and *α*9 *β*1 integrin on the LEC surface. Stimulation of VEGFR-3 transmits potent mitogenic signals into LECs, whereas activation of integrin induces redistribution of the cellular actin cytoskeleton, leading to change in cell shape and formation of pseudopodia enabling cells to migrate eventually new lymphangiogenesis.

When processed through proteolysis in the ECM to mature forms, VEGF-C has been reported to also activate VEGFR-2 on the surface of the BECs, thereby inducing angiogenesis (Joukov *et al*, 1996). Such effects have been reported in experimental studies (Oh *et al*, 1997), but has not been fully studied in human breast cancer. Unlike a recent report by Hu *et al* (2005), the present study indicates that tumours with high expression of VEGF-C are significantly associated with a higher MVD, suggesting that VEGF-C has an additional angiogenic effect in breast cancer and may reflect active proteolysis found in the tumour microenvironment. The study by Hu and co-workers found no such association, however, such differences may reflect the different patient population and the smaller number of specimens in the study by Hu.

Similar to other studies (Nakamura *et al*, 2003b), when high expression of VEGF-C was examined in relation to the clinicopathological characteristics and to patient survival, a significant positive relationship was found with the presence of both LN metastasis (*P*<0.001) and distant metastasis (*P*=0.008). High expression was also significantly associated with the occurrence of regional recurrence (*P*=0.025), and poorer OS (*P*=0.028), perhaps due to the potent ability of VEGF-C to induce both lymphangiogenesis and angiogenesis, thereby providing open channels for dissemination of malignant cells.

Tumours which express high levels of both VEGF-A and -C were found to have the worst prognosis when compared with tumours expressing low levels of both factors or tumours expressing high levels of either of the growth factors individually. These results support the notion that the two VEGF family members may be involved in tumour progression at two interrelated steps. Evaluation of expression of both VEGF-A and -C may be an important factor to identify breast cancer patients at higher risk of recurrence and in need of adjuvant therapy.

VEGF-D is a relatively recent member of the VEGF family that shares some structural and functional properties with VEGF-C (Yamada *et al*, 1997). In experimental animal tumour models, overexpression of VEGF-D was found to be significantly associated with higher rate of LN metastasis, increased tumour angiogenesis and larger tumour sizes (Stacker *et al*, 2001; Von Marschall *et al*, 2005; Yonemura *et al*, 2005). However, in human tumours, there is variation amongst results. It has been reported to be significantly associated with high LVD, LN metastasis and poorer survival in gastric (Shida *et al*, 2005), endometrial (Yokoyama *et al*, 2003) and colorectal carcinomas (White *et al*, 2002). In head and neck tumours (O-Charoenrat *et al*, 2001), such relationships have not been found. In other studies in breast and in lung carcinoma, low levels of expression of VEGF-D was associated with poorer prognosis (Niki *et al*, 2000; Koyama *et al*, 2003). In the current study, there was a trend for tumours with higher VEGF-D expression to have a higher rate of LN metastasis, regional recurrence and distant metastasis, however, none of these reached statistical significance. Similar findings were reported by Yang *et al* (2002). In addition, no significant association was found between expression of VEGF-D and either LVD or MVD. In a study on breast cancer, a significant association was found between high VEGF-D gene expression, using real-time polymerase chain reaction and LVD (Choi *et al*, 2005). Such differences in results may be due to the different methods in assessment of VEGF-D expression. Although VEGF-D showed lymphangiogenic and angiogenic effects in xenografted tumours, its biological role in human breast cancer is still unclear and is in need of further study. It is probable that VEGF-D plays different roles in different tumour types.

In conclusion, it appears from this study of human breast cancers that, as has been reported by *in vitro* studies, VEGF-A plays a role in lymphangiogenesis. It also appears that breast cancers, which express high levels of VEGF-A and -C are characterised by greater angiogenesis and lymphangiogenesis and are associated with the presence of both LN and distant metastasis. Such tumours behave more aggressively as indicated by the associations with shorter DFI and OS. Both growth factors appear to play an important role in the progression of breast carcinoma and to have a significant impact on patient prognosis. Examination of expression of either VEGF-A and -C can be used to identify a subset of breast cancer at higher risk for development of recurrence and distant metastasis, with the recommendation that both be assessed to further delineate those at highest risk.

## Figures and Tables

**Table 1:** Relationship between expression of VEGF-A, VEGF-C and VEGF-D

	**VEGF-C no. (%)**			**VEGF-D no. (%)**		
	**Low**	**High**	***P*-value**	**Low**	**High**	***P*-value**
*VEGF*-A						
Low	75 (71)	31 (28)		61 (57)	45 (42)	
High	39 (55)	32 (45)	**0.046**	35 (49)	36 (51)	0.354
						
*VEGF*-C						
Low				73 (64)	41 (36)	
High				23 (36)	40 (64)	**0.001**

VEGF-A=vascular endothelial cell growth factor-A; VEGF-C=vascular endothelial cell growth factor-C. Bold values denotes statistical significance.

**Table 2:** Association between expression of VEGFs with clinicopathological criteria, lymphangiogenesis and angiogenesis

	**No. of patients**	**No. (%) of VEGF-A high-expressing specimens**	***P*-value**	**No. (%) of VEGF-C high-expressing specimens**	***P*-value**	**No. (%) of VEGF-D high-expressing specimens**	***P*-value**
*Age*							
<50 years	51	18 (35)		15 (29)		23 (45)	
>50 years	126	53 (42)	NS	48 (38)	NS	58 (46)	NS
*Size*							
<1.5 cm	65	19 (29)		16 (25)		28 (43)	
>1.5 cm	112	52 (46)	**0.038**	47 (42)	**0.023**	53 (47)	NS
*Grade*							
I	45	9 (20)		9 (20)		22 (49)	
II	62	19 (31)		29 (47)		30 (48)	
III	70	43 (60)	**<0.001**	25 (36)	NS	29 (41)	NS
*LN status*							
Negative	125	36 (29)		31 (25)		53 (42)	
Positive	52	35 (76)	**<0.001**	32 (61)	**<0.001**	28 (54)	NS
*NPI*							
Good	74	16 (22)		19 (26)		33 (45)	
Intermediate	88	42 (47)		36 (41)		41 (47)	
Poor	15	13 (87)	**<0.001**	8 (53)	**0.012**	7 (47)	NS
*ER*							
Negative	56	37 (64)		19 (34)		28 (50)	
Positive	112	34 (30)	**<0.001**	41 (37)	NS	49 (44)	NS
*PR*							
Negative	72	41 (55)		27 (38)		32 (44)	
Positive	93	30 (32)	**0.004**	31 (33)	NS	42 (45)	NS
*RR*							
No	136	55 (40)		42 (31)		59 (43)	
Definite	41	16 (38)	NS	21 (51)	**0.025**	22 (54)	NS
*MVD*							
Low	101	29 (29)		31 (31)		48 (48)	
High	76	42 (55)	<0.001*	32 (41)	0.002*	33 (43)	NS^*^
*LVD*							
Low	122	42 (35)		36 (29)		56 (46)	
High	55	29 (51)	0.013*	27 (49)	0.014*	25 (46)	NS^*^
*LVI*							
Absent	108	36 (33)		33 (31)		48 (44)	
Definite	69	35 (52)	**0.028**	30 (44)	**0.107**	33 (48)	NS
*DM*							
No	156	58 (37)		51 (33)		72 (45)	
Definite	18	13 (71)	**0.010**	12 (67)	**0.008**	9 (50)	NS

DM=distant metastasis; ER=oestrogen receptor; LN=lymph node; LVD=lymph vessel density; MVD=microvessel density; NPI=Nottingham prognostic index; NS=nonsignificant; PR=progesterone receptor, RR=regional recurrence.

Statistical significance was tested using 2 × 2 table and *χ*^2^ test, except (^*^), where it was performed using Mann–Whitney test. Bold values denotes statistical significance.
